# Gradient and scattering forces of anti-reflection-coated spheres in an aplanatic beam

**DOI:** 10.1038/s41598-018-35575-1

**Published:** 2018-11-27

**Authors:** Neng Wang, Xiao Li, Jun Chen, Zhifang Lin, Jack Ng

**Affiliations:** 1 Department of Physics, Hong Kong Baptist University, Hong Kong, China; 2 Department of Physics, Hong Kong University of Science and Technology, Hong Kong, China; 3 Institute of Theoretical Physics and Collaborative Innovation Center of Extreme Optics, Shanxi University, Shanxi, 030006 China; 4 Department of Physics, Fudan University, Shanghai, 200433 China; 5 Institute of Computational and Theoretical Studies, Hong Kong Baptist University, Hong Kong, China

## Abstract

Anti-reflection coatings (ARCs) enable one to trap high dielectric spheres that may not be trappable otherwise. Through rigorously calculating the gradient and scattering forces, we directly showed that the improved trapping performance is due to the reduction in scattering force, which originates from the suppression of backscattering by ARC. We further applied ray optics and wave scattering theories to thoroughly understand the underlying mechanism, from which, we inferred that ARC only works for spherical particles trapped near the focus of an aplanatic beam, and it works much better for large spheres. For this reason, in contradiction to our intuition, large ARC-coated spheres are sometimes more trappable than their smaller counter parts. Surprisingly, we discovered a scattering force free zone for a large ARC-coated sphere located near the focus of an aplanatic beam. Our work provides a quantitative study of ARC-coated spheres and bridges the gap between the existing experiments and current conceptual understandings.

## Introduction

**Figure 1:**
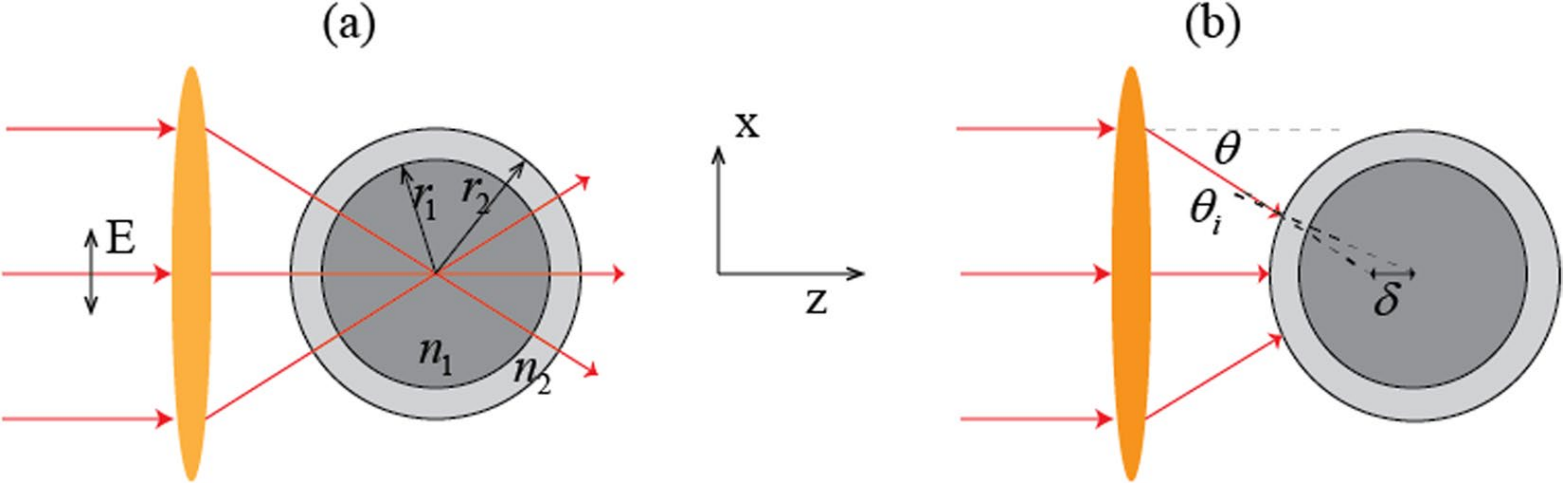
Schematic illustration of an ARC-coated sphere located (**a**) at and (**b**) off the focus of an aplanatic beam.

Optical tweezers^1–4^, which traps small particles using a tightly focused laser beam, have found fruitful applications across various scientific areas, ranging from physics^5–8^ to chemistry^9,10^, and to biology^11,12^. Particles in a laser beam are subject to both the optical scattering force **F**_*s*_(**x**) and gradient force **F**_*g*_(**x**)^13–18^. The former is divergence-less (∇ · **F**_*s*_ = 0) and tends to axially push the particle away from the focus, while the latter is curl-less (∇ × **F**_*g*_ = 0) and tends to attract the particle towards the focus. A particle can be trapped if the gradient force dominates^2^. Thus, optical trapping can be improved by either reducing the scattering force or enhancing the gradient force. So far, these two aspects are realized by either shaping the beam^19–22^ or customizing the morphology of the trapped object^23,24^. A simple and elegant example is to coat an anti-reflection coating (ARC) on a high dielectric spherical particle^25–27^, as shown in Fig. 1. While a bare high dielectric particle is highly reflective and thus not trappable, the ARC makes it trappable. Interestingly, such trapped ARC coated particle possesses a large transverse gradient force (up to a nano-Newton), which opens up new potentials for applications, such as protein unfolding, amyloid fibril disruption, cell adhesion and contraction^26^. It is commonly believed that ARC can reduce the scattering force, however, it was not rigorously proven. The difficulty partly lies in the fact that one cannot easily obtain the gradient and scattering force for Mie sized particles. Moreover, although the backward scattering is believed to be suppressed for ARC-coated particles, the mechanism was not well explained, especially from the perspective of wave scattering theory.

Here, we reinvestigate the optical trapping of ARC-coated spheres by explicitly calculating their gradient and scattering forces using the approach adopted by ref.^18^, and studying their scattering properties when illuminated by an aplanatic beam or a plane wave. The numerical results show clearly that the scattering force is reduced by several orders in magnitude near the focus when the sphere is coated by ARC, which directly verifies the conclusions in the previous work^25^. The scattering force is reduced due to the suppression of the backward scattering, which can be seen clearly from the scattering features. By analytically studying the Mie coefficients of the ARC-coated sphere, we find that the low order Mie coefficients satisfy the generalized Kerker condition that leads to the suppression of the backward scattering^28,29^. However, because the high order Mie coefficients do not satisfy the condition, the backward scattering can be suppressed only when the scattering is dominated by the low order scattering coefficients, which requires the incident beam to be an aplanatic beam whose beam shape coefficient decays as its order grows, but not a plane wave whose beam shape coefficient increases with its order. Also, the generalized Kerker condition is fulfilled for more orders as the particle size increases, implying that ARC works much better for the larger size spheres.

## Results

### The scattering and gradient forces acting on uncoated and ARC-coated spheres

Consider the ARC-coated sphere shown in Fig. 1. It has inner and outer radii *r*_1_ and *r*_2_, respectively. Its core, coating, and surrounding have refractive indices of *n*_1_, *n*_2_, and *n*_0_, respectively. The ARC that coated on the sphere is similar to that of a planar surface, which is characterized by a refractive index ${n}_{2}\approx \sqrt{{n}_{1}{n}_{0}}$ and a thickness (*r*_2_ − *r*_1_) = *λ*/(4*n*_2_), where *λ* is the vacuum wavelength. We consider the same materials as in ref.^26^, namely, *n*_0_ = 1.33 (water), *n*_1_ = 2.3 (anatase titania), and *n*_2_ = 1.78 (amorphous titania), which approximately fulfill the ARC conditions^30^. The trapping beam is an *x*-polarized and *z*-propagating fundamental Gaussian beam with vacuum wavelength *λ* = 1.064 μm and is focused by a high numerical aperture (NA) objective lens with NA = 1.3, if not otherwise stated. As shown in Fig. 1, the origin of the coordinate system is chosen to be the beam focus.

The total optical force **F**(**x**) acting on the sphere can be calculated rigorously using the generalized Lorenz-Mie theory and the Maxwell stress tensor^31–34^. Such an approach makes no approximation within classical electrodynamics and is subject only to the numerical truncation error. The total force can then be numerically decomposed into the scattering force **F**_*s*_(**x**) and gradient force **F**_*g*_(**x**) using the approach presented in ref.^18^:

1 $$\begin{array}{c}{{\bf{F}}}_{s}({\bf{x}})=\int \frac{[{\bf{q}}\times \tilde{{\bf{F}}}({\bf{q}})]\times {\bf{q}}/{q}^{2}}{{(2\pi )}^{3/2}}{e}^{i{\bf{q}}\cdot {\bf{x}}}{d}^{3}{\bf{q}},\\ {{\bf{F}}}_{g}({\bf{x}})=\int \frac{[{\bf{q}}\cdot \tilde{{\bf{F}}}({\bf{q}})]\cdot {\bf{q}}/{q}^{2}}{{(2\pi )}^{3/2}}{e}^{i{\bf{q}}\cdot {\bf{x}}}{d}^{3}{\bf{q}},\end{array}$$

where $\tilde{{\bf{F}}}({\bf{q}})$ is the Fourier transform of the total optical force, which is given by $\tilde{{\bf{F}}}({\bf{q}})=\int \frac{{\bf{F}}({\bf{x}})}{{(2\pi )}^{3/2}}{e}^{-i{\bf{q}}\cdot {\bf{x}}}{d}^{3}{\bf{x}}.$

**Figure 2:**
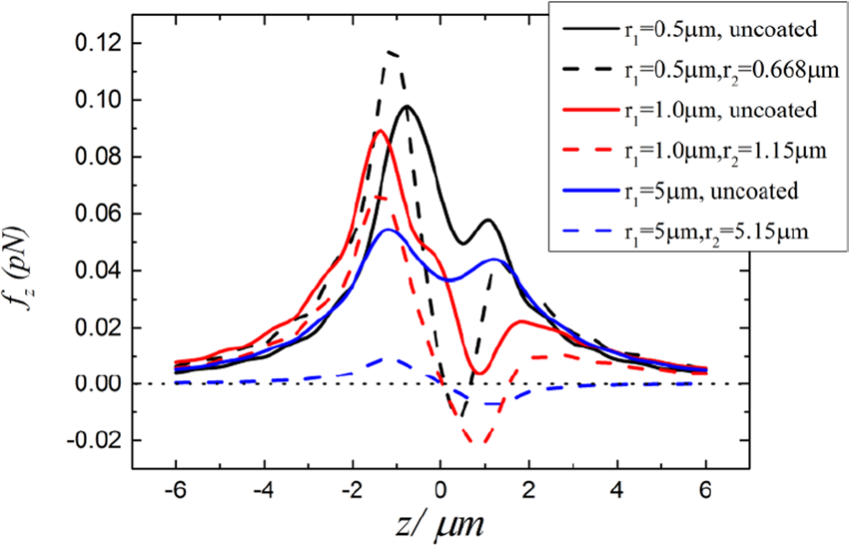
The longitudinal optical forces acting on the uncoated (solid lines) and ARC-coated (dotted lines) spheres in a Gaussian beam. The spheres are located on the beam axis, and *E*_0 _= 10^6^*V*/*m*.

**Figure 3:**
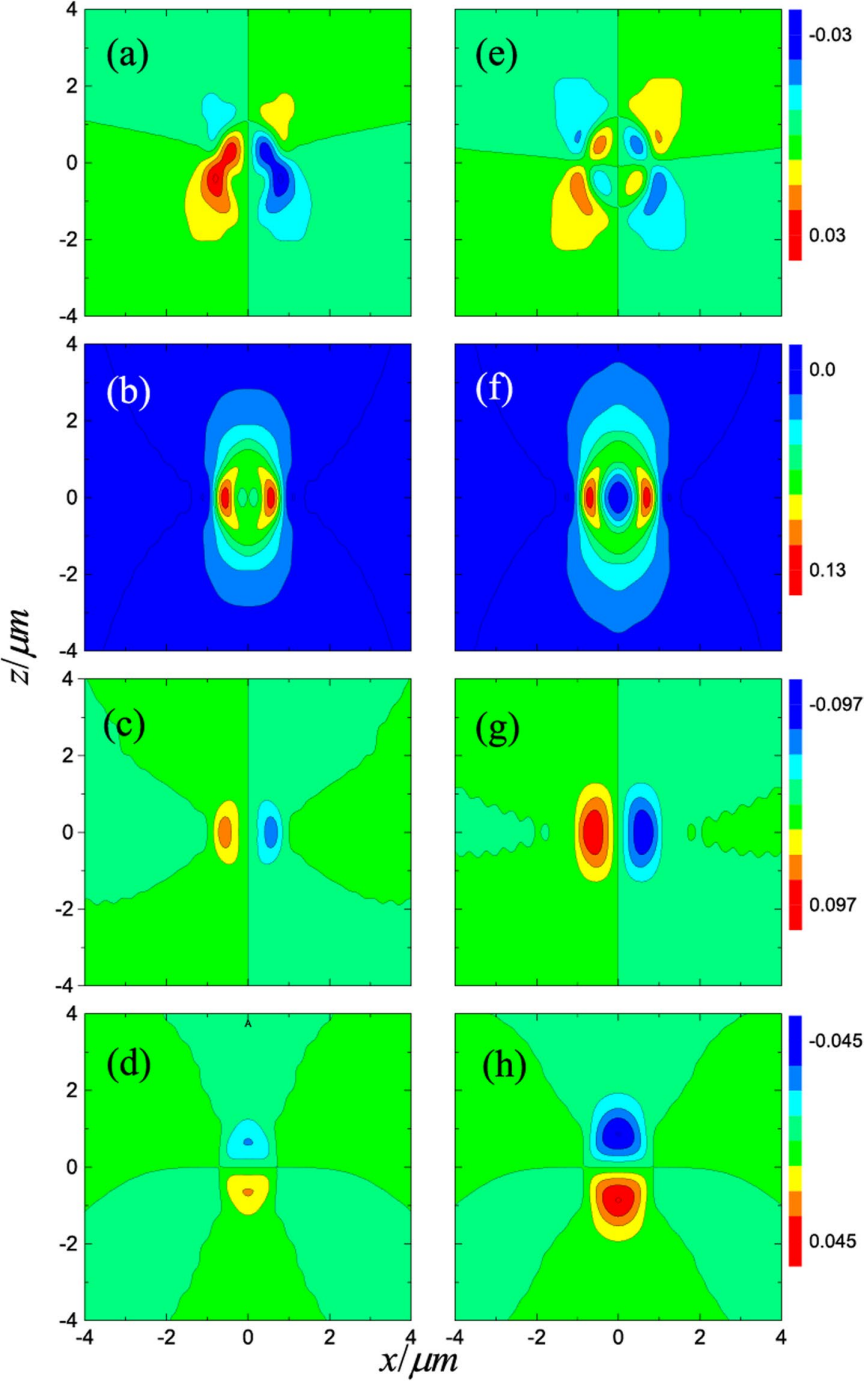
Gradient and scattering force for uncoated (left column) and ARC-coated (right column) spheres illuminated by an *x*-polarized Gaussian beam. The first, second, third, and fourth rows are, respectively, (**F**_*s*_)_*x*_, (**F**_*s*_)_*z*_, (**F**_*g*_)_*x*_, and (**F**_*g*_)_*z*_. The inner and outer radii of the sphere are *r*_1_ = 0.5 μm and *r*_2_ = 0.668 μm, respectively.

**Figure 4:**
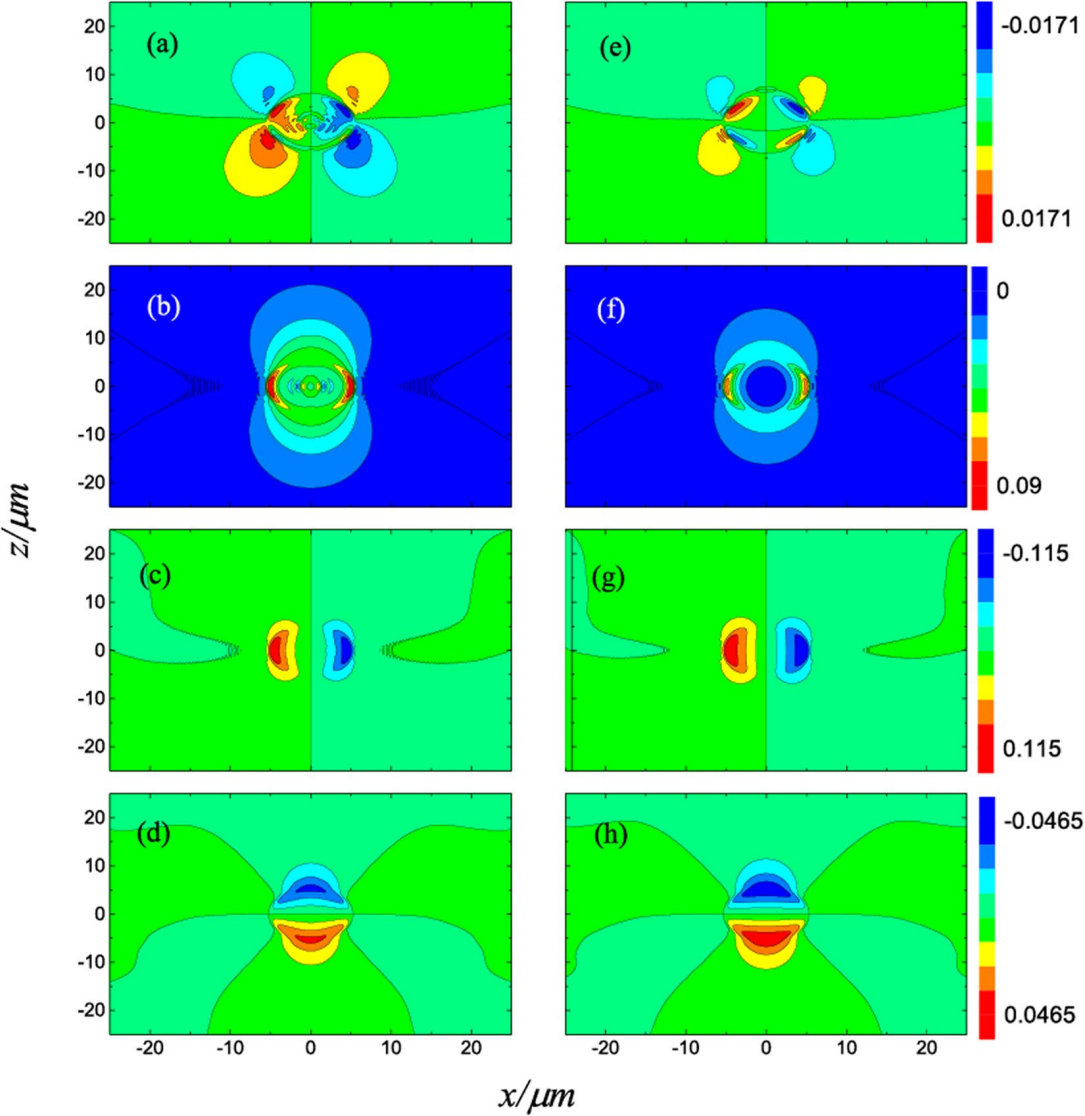
Gradient and scattering force for an uncoated (left column) and ARC-coated (right column) spheres illuminated by an *x*-polarized Gaussian beam. The first, second, third, and fourth rows are, respectively, (**F**_*s*_)_*x*_, (**F**_*s*_)_*z*_, (**F**_*g*_)_*x*_, and (**F**_*g*_)_*z*_. The unit of optical force is pN. The inner and outer radii of the sphere are *r*_1_ = 5.0 μm and *r*_2_ = 5.15 μm, respectively. Here, *E*_0_ = 10^6^*V*/*m*.

**Figure 5:**
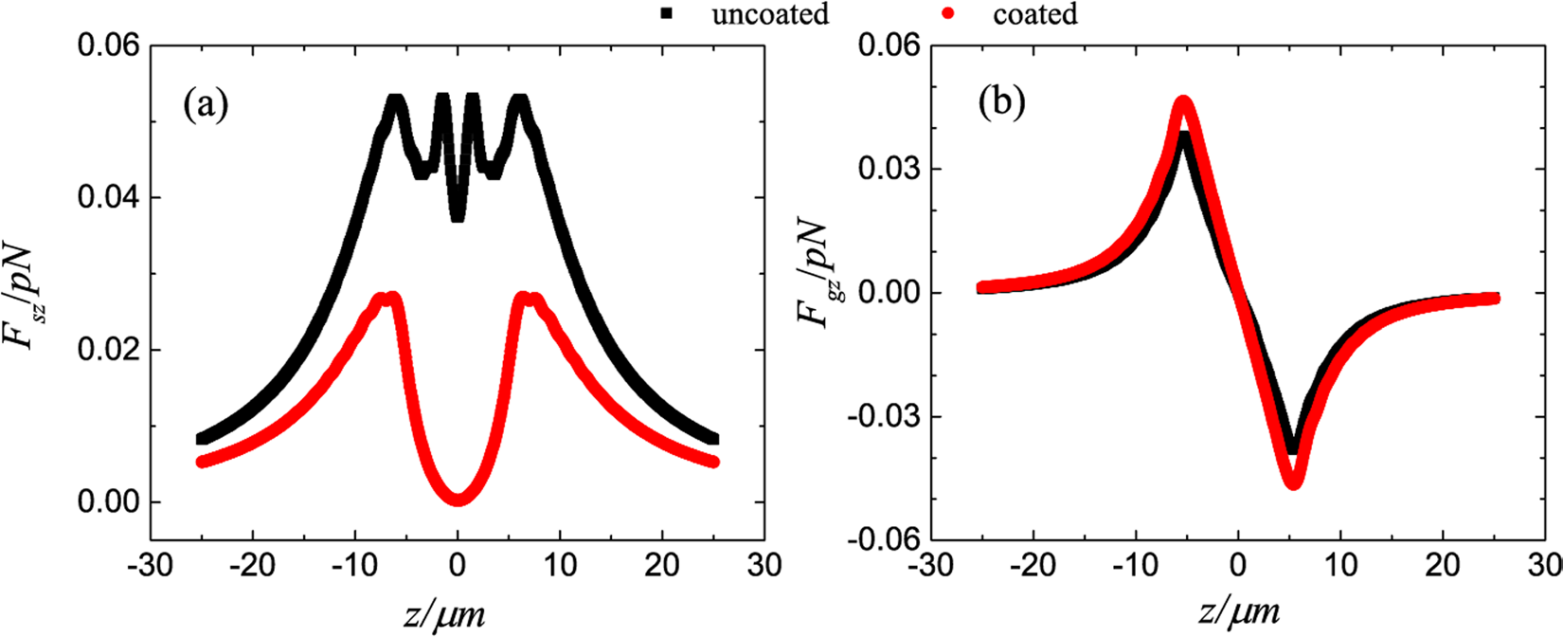
The longitudinal component of scattering (**a**) and gradient (**b**) forces for uncoated (black) and ARC-coated (red) spheres. The parameters used are the same as in Fig. 4.

In Fig. 2, we plot the longitudinal (*z* direction) optical forces acting on uncoated (solid lines) and ARC-coated (dotted lines) spheres along the beam axis. Trapping equilibrium does not exist for the uncoated spheres, while stable axial trapping is possible after introducing the ARC, in agreement with refs^25–27^. The gradient and scattering forces on the *xz*-plane for spheres with and without the ARC are plotted in Fig. 3. Comparing the scattering force for the uncoated and coated spheres, i.e. comparing Fig. 3(a) with Fig. 3(e), and comparing Fig. 3(b) with Fig. 3(f), one can clearly see that the ARC significantly reduces the scattering force, especially near the focus as shown in the center blue spot in Fig. 3(f). Similarly, comparing Fig. 3(c and g), and comparing Fig. 3(d,h), it is clear that the gradient forces are enhanced. This is expected, as the particle size increases after coating. For a *r*_1_ = 5.0 μm sphere, the scattering and gradient forces are shown in Fig. 4. The scattering force is also greatly reduced, however, the gradient force is only enhanced slightly after coating, as illustrated in Fig. 5. For large particles, the relative volume of the ARC is smaller, therefore the enhancement in gradient force is also small. In short, ARC-coated spheres are trappable because of both the reduction in scattering force and enhancement in gradient force. As shown in Figs 3(f), 4(f) and 5(a), the scattering force is significantly diminished near the focus, especially for the larger particles. This is because the ARC effectively eliminated the reflection for a big sphere located at the focus. Intererstingly, this creates a zone where the conservative force dominates, which is useful when the scattering force is undesirable.

The scattering force is vanishing at the beam focus for the coated particle shown in Fig. 4(f). For this reason, the ARC-coated sphere is trapped close to the beam center, where the transverse gradient force reaches its maximum. This explains why a large trapping force is realized^26^. First, the gradient force is enhanced, mostly due to the increase in particle size. Second, the reduction of scattering force drives the equilibirum towards the focus, where the transverse gradient force is largest.

### The working mechanism of ARC

The working mechanism of ARC in the large particle limit, can be captured by the ray optics, where the beam is decomposed into a bunch of rays^13^, as illustrated in Fig. 1(a). For a particle located at the focus, all incident rays are normal to the sphere’s surface. If the particle is sufficiently large such that the curvature of the sphere can be ignored, the rays will be fully transmitted without reflection, just as for a planar surface. The absence of reflection significantly reduces the longitudinal scattering force. However, when the particle is not at the focus, as illustrated in Fig. 1(b), the rays and the surface normal are not parallel, and the angle between them varies with position. Consequently, no ARC can be consistently matched to the position-dependent angle. Accordingly, the backward scattering is not suppressed, and the mechanism for reducing the scattering force no longer works. One can then infer that the ARC only works for an aplanatic beam trapping a particle near the focus, and not for other types of incident field such as a plane wave. We note that using high index dielectrics as the ARC can mitigate the problem^35,36^. By the Snell’s law, the refraction angle is small for high index coating. The rays inside the coating are almost normal to the surfaces, allowing the reflection to be suppressed by interference. Accordingly, ARC with a higher index works better for off-focus spheres, which also extends the scattering force free zone.

The ray optics theory is simple and elegant, yet it is inaccurate for small particles. Hereafter, we will apply a rigorous wave scattering theory. We introduce the normalized scattering intensity, which can describe the features of scattering^37,38^:

2 $$S(\theta ,\varphi )=\mathop{\mathrm{lim}}\limits_{kr\to \infty }{(kr)}^{2}\frac{|{{\bf{E}}}_{s}(r,\theta ,\varphi ){|}^{2}}{|{E}_{0}{|}^{2}},$$

where (*r*, *θ*, *ϕ*) is the spherical coordinate system concentric with the particle, *k* is the wavenumber in the medium, and **E**_*s*_(*r*, *θ*, *ϕ*) is the scattered electric field. We shall only focus on the backward scattering (characterized by *θ* = *π*) for a particle located on the beam axis, where Eq. (2) has a succinct analytic expression (see detail in the method part):

3 $$S(\pi ,\varphi )={|\sum _{n=1}^{\infty }(n+1){g}_{n}({a}_{n}-{b}_{n})(\cos \varphi {{\bf{e}}}_{\theta }-\sin \varphi {{\bf{e}}}_{\varphi })|}^{2},$$

where *a*_*n*_ and *b*_*n*_ are the Mie coefficients of the coated sphere, and

4 $${g}_{n}=\frac{\sqrt{2n+1}}{2{i}^{n}}[\frac{(n+1)i}{2n+1}{j}_{n-1}(ik{z}_{c})+{j}_{n}(ik{z}_{c})-\frac{ni}{2n+1}{j}_{n+1}(ik{z}_{c})]\frac{k{l}_{0}}{{e}^{k{l}_{0}}},$$

are the beam shape coefficients of the Gaussian beam^39^, where ${l}_{0}=1/2k{w}_{0}^{2}$ is the Rayleigh diffraction length, *w*_0_ = *n*_0_*λ*/(*π*⋅*NA*) is the waist radius, and *z*_*c*_ = *l*_0_ + *iz* with *z* being the location of the sphere.

The Mie coefficients of the coated sphere are^25,26^

5 $$\begin{array}{c}{a}_{n}=\frac{{\psi }_{n}(y)[{\psi }_{n}\text{'}({\tilde{n}}_{2}y)-{A}_{n}{\chi }_{n}\text{'}({\tilde{n}}_{2}y)]-{\tilde{n}}_{2}{\psi }_{n}\text{'}(y)[{\psi }_{n}({\tilde{n}}_{2}y)-{A}_{n}{\chi }_{n}({\tilde{n}}_{2}y)]}{{\xi }_{n}(y)[{\psi }_{n}\text{'}({\tilde{n}}_{2}y)-{A}_{n}{\chi }_{n}\text{'}({\tilde{n}}_{2}y)]-{\tilde{n}}_{2}{\xi }_{n}\text{'}(y)[{\psi }_{n}({\tilde{n}}_{2}y)-{A}_{n}{\chi }_{n}({\tilde{n}}_{2}y)]},\\ {b}_{n}=\frac{{\tilde{n}}_{2}{\psi }_{n}(y)[{\psi }_{n}\text{'}({\tilde{n}}_{2}y)-{B}_{n}{\chi }_{n}\text{'}({\tilde{n}}_{2}y)]-{\psi }_{n}\text{'}(y)[{\psi }_{n}({\tilde{n}}_{2}y)-{B}_{n}{\chi }_{n}({\tilde{n}}_{2}y)]}{{\tilde{n}}_{2}{\xi }_{n}(y)[{\psi }_{n}\text{'}({\tilde{n}}_{2}y)-{B}_{n}{\chi }_{n}\text{'}({\tilde{n}}_{2}y)]-{\xi }_{n}\text{'}(y)[{\psi }_{n}({\tilde{n}}_{2}y)-{B}_{n}{\chi }_{n}({\tilde{n}}_{2}y)]},\\ \,{A}_{n}=\frac{{\tilde{n}}_{2}{\psi }_{n}({\tilde{n}}_{2}x){\psi }_{n}\text{'}({\tilde{n}}_{1}x)-{\tilde{n}}_{1}{\psi }_{n}\text{'}({\tilde{n}}_{2}x){\psi }_{n}({\tilde{n}}_{1}x)}{{\tilde{n}}_{2}{\chi }_{n}({\tilde{n}}_{2}x){\psi }_{n}\text{'}({\tilde{n}}_{1}x)-{\tilde{n}}_{1}{\chi }_{n}\text{'}({\tilde{n}}_{2}x){\psi }_{n}({\tilde{n}}_{1}x)},\\ \,{B}_{n}=\frac{{\tilde{n}}_{2}{\psi }_{n}({\tilde{n}}_{1}x){\psi }_{n}\text{'}({\tilde{n}}_{2}x)-{\tilde{n}}_{1}{\psi }_{n}\text{'}({\tilde{n}}_{1}x){\psi }_{n}({\tilde{n}}_{2}x)}{{\tilde{n}}_{2}{\chi }_{n}\text{'}({\tilde{n}}_{2}x){\psi }_{n}({\tilde{n}}_{1}x)-{\tilde{n}}_{1}{\chi }_{n}({\tilde{n}}_{2}x){\psi }_{n}\text{'}({\tilde{n}}_{1}x)},\end{array}$$

where ${\psi }_{n}(x)=x{j}_{n}(x),{\chi }_{n}(x)=-\,x{y}_{n}(x),{\xi }_{n}(x)=x{h}_{n}^{(1)}(x)$ are Ricatti-Bessel functions, *x* = *kr*_1_ and *y* = *kr*_2_ are dimensionless size parameters for the core and shell, respectively, and ${\tilde{n}}_{1}$ = *n*_1_/*n*_0_ and ${\tilde{n}}_{2}$ = *n*_2_/*n*_0_ are normalized refractive indices. For ARC-coated spheres, ${\tilde{n}}_{1}={\tilde{n}}_{2}^{2}$ and 2${\tilde{n}}_{2}$(*y* − *x*) = *π*. We first consider the large particle limit with *x* → ∞. Here, the asymptotical formulae for the Ricatti-Bessel functions are introduced:

6 $$\begin{array}{c}{\xi }_{n}(x)={\psi }_{n}(x)-i{\chi }_{n}(x)\sim {(-i)}^{n+1}{e}^{ix},\\ \,{\xi }_{n}\text{'}(x)={\psi }_{n}\text{'}(x)-i{\chi }_{n}\text{'}(x)\sim {(-i)}^{n}{e}^{ix}.\end{array}$$

Using these expressions, the Mie coefficients reduce to a simple expression

7 $${a}_{n}={b}_{n}=\frac{\sin \,y+\,\cot ({\tilde{n}}_{1}x)\cos \,y}{\cot ({\tilde{n}}_{1}x)-i}{e}^{-iy}.$$

Eq. (7) indicates that for a very large ARC-coated sphere, the Mie coefficients of all orders are the same. According to Eq. (3), the backward scattering becomes zero. For a sphere with finite size, although Eq. (7) is not exact, low order Mie coefficients can still be approximated by Eq. (7) and fulfill *a*_*n*_ ≈ *b*_*n*_, which can be seen from the numerical examples shown in Tables 1 and 2. Meanwhile, for the higher order terms, we note that for the Gaussian beam, (*n* + 1)*g*_*n*_ decreases rapidly with *n*, so the contributions from the high order terms in Eq. (3) are not important. Because the low order Mie coefficients satisfy *a*_*n*_ ≈ *b*_*n*_, the total backward scattering intensity almost vanishes: see Eq. (3). One could have reached a similar conclusion by noting that an impedance-matched homogeneous sphere, with its permittivity equal to its permeability, will have *a*_*n*_ = *b*_*n*_, and its reflection is expected to be low. The condition *a*_*n*_ = *b*_*n*_ is also called the generalized Kerker condition which leads to the directional emission^28,29^. To achieve the Kerker condition, particles fabricated are usually complex in structures and the frequency is tuned to support mutiple resonances simultaneously^29,40–43^. However, these methods are technically complicated, and the generalized Kerker condition is fulfilled only for a few orders. It is interesting to see that by coating an ARC on a large size sphere, many multiople orders will fulfill this condition without the need of resonances. If the scattering is dominated by the low orders which satisfy the generalized Kerker condition, unidirectional emmison is achieved. We can see that the ARC-coated large spheres have the potential for manipulating specific light fields.

**Table 1:** Mie coefficients for an ARC-coated sphere with inner and outer radii *r*_1_ = 1.0 μm and *r*_2_ = 1.15 μm, respectively. The sphere is located at the focus of a linearly polarized Gaussian beam. The Mie coefficient calculated by Eq. (7) is 0.0272428 + 0.16279*i*.

*n*	*a*_*n*_	*b*_*n*_	(*n* + 1)*g*_*n*_
1	0.036436 + 0.187373i	0.0487883 + 0.215425 i	1.32691
2	0.101541 + 0.302044i	0.0802716 + 0.271713 i	1.53799
3	0.186108 + 0.389194i	0.223248 + 0.416423 i	1.16625
4	0.417332 + 0.493119i	0.360582 + 0.480169i	0.655511
5	0.656048 + 0.475025i	0.69021 + 0.462407i	0.291314
6	0.93799 + 0.241173i	0.962933 + 0.188926i	0.106639
7	0.961466 − 0.192481i	0.999996 + 0.00189196i	0.0330921

**Table 2:** Mie coefficients for an ARC-coated sphere with inner and outer radii *r*_1_ = 5.0 μm and *r*_2_ = 5.15 μm, respectively. The sphere is located at the focus of a linearly polarized Gaussian beam. The Mie coefficient calculated by Eq. (7) is 0.452864 - 0.497773*i*.

*n*	*a*_*n*_	*b*_*n*_	(*n* + 1)*g*_*n*_
1	0.455965 − 0.498057i	0.482446 − 0.499692i	1.3392 − 0.0705567i
2	0.461564 − 0.49852i	0.43433 − 0.495669i	1.56435 − 0.232702i
3	0.402758 − 0.490453i	0.431044 − 0.495222i	1.17898 − 0.330319i
4	0.389207 − 0.48757i	0.360061 − 0.480018i	0.642546 − 0.286775i
5	0.310372 − 0.462646i	0.340283 − 0.473804i	0.267418 − 0.17560i
6	0.279918 − 0.448959 i	0.250415 − 0.433252i	0.0874659 − 0.0819216i
7	0.190427 − 0.392638i	0.218725 − 0.413382i	0.0226966 − 0.0305472i

**Figure 6:**
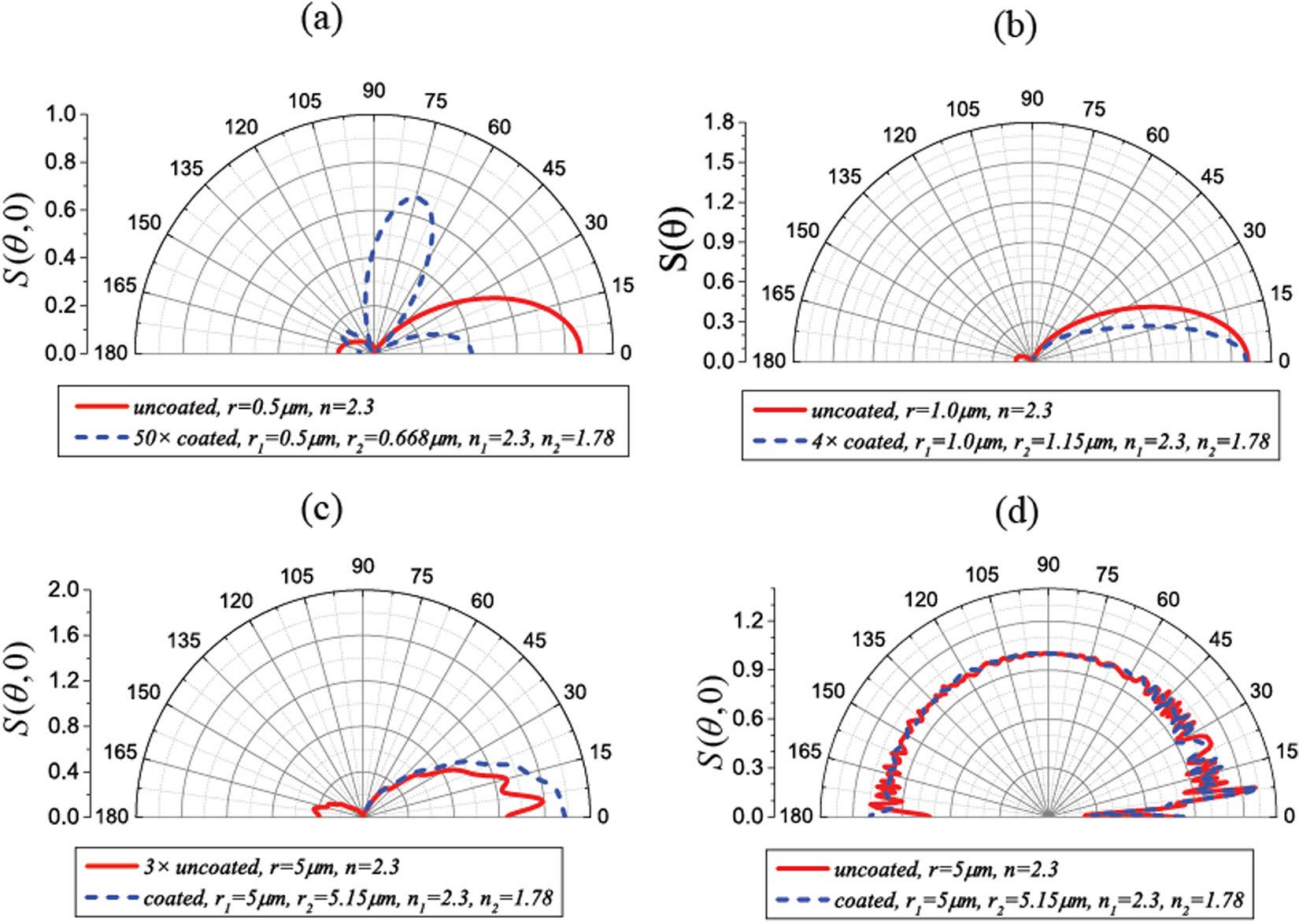
Normalized scattering intensities for the uncoated (red solid lines) and ARC-coated (blue dashed lines) spheres with different sizes in a Gaussian beam (**a**–**c**) and a plane wave (**d**).

Figure 6(a–c) plots the normalized scattering intensity for coated (blue dashed lines) and uncoated (red solid lines) spheres located at the Gaussian beam focus. Clearly, the uncoated high dielectric spheres have non-vanishing backward scatterings, leading to a strong forward scattering force. By applying ARCs on the spheres, though the total scattering efficiencies are not necessarily enhanced or reduced (see the amplitude of the scattering intensity), the backward scatterings are suppressed. In contrast, the ARC is not working for the plane wave incident case, as shown in Fig. 6(d). Because $(n+1){g}_{n}=(n+1)\sqrt{n+1}/2$ for the plane wave does not vanish for high orders, where the generalized Kerker condition is not fulfilled, the backward scattering cannot be suppressed.

### ARC works better for large spheres

**Figure 7:**
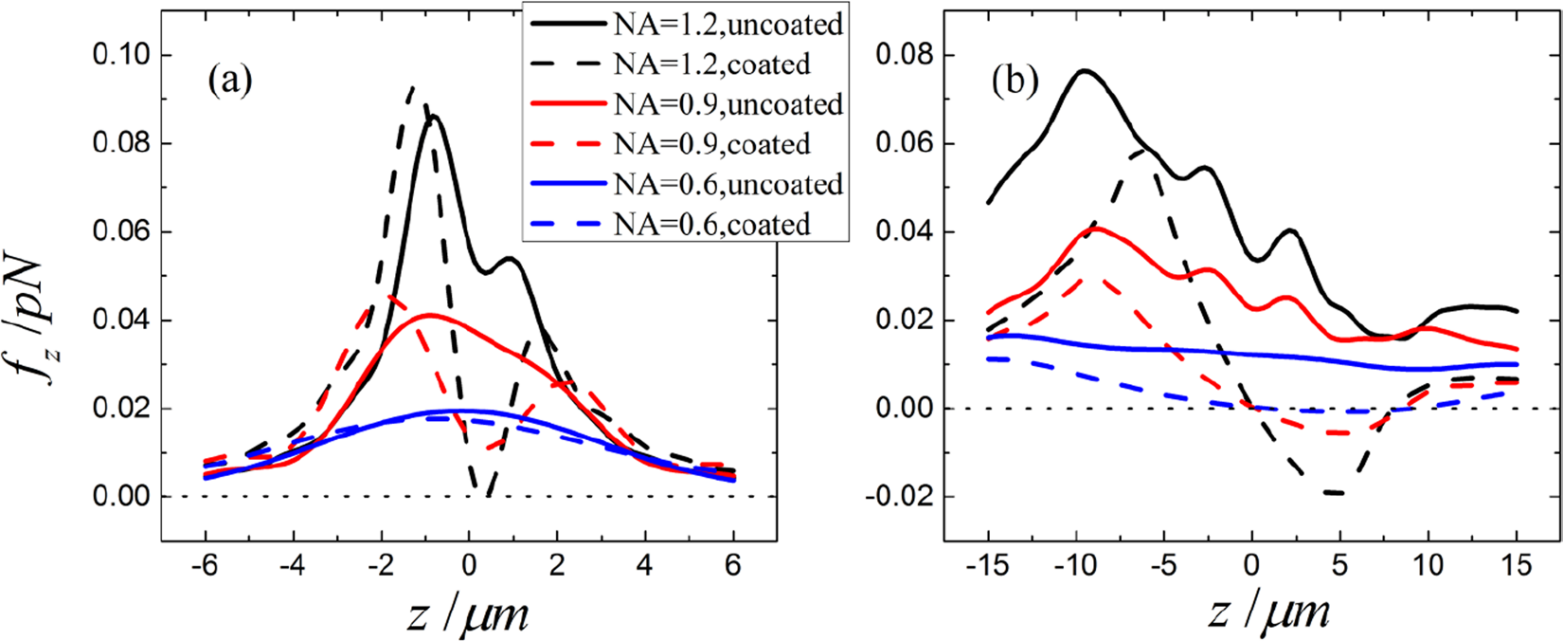
Axial optical forces for beams with different NA. (**a**) is for a particle with core radius 0.5 μm, and (**b**) is for a particle with core radius 5.0 μm.

The axial forces for spheres illuminated by Gaussian beams with different NA are plotted in Fig. 7. For the 0.5-micron radius particle (vaccum wavelength is *λ* = 1.064 μm), a large NA is required for trapping even with the ARC, as shown in Fig. 7(a). In contrast, for the case of 5-micron radius partilce shown in Fig. 7(b), even for a small NA, the ARC can effectively reduces the forward forces, making the high dielectric particle trappable. Therefore, the ARC wokrs better for the larger size spheres. This seems to oppose our intuition that smaller particles are easier to trap, as the ratio of the gradient force to scattering force is much more favorable. Using Eq. (3), we can explain why the ARC works better for large spheres. For smaller NA, (*n* + 1)*g*_*n*_ decreases slower as *n* grows, so that higher order Mie coefficients will contribute to the backward scattering. As fewer multipole orders fulfill *a*_*n*_ ≈ *b*_*n*_ for smaller spheres, while more orders do so for larger spheres, the ARC reduces the backward scattering for larger spheres more efficiently: see also Fig. 6(a,c).

## Conclusions

In summary, we have thoroughly addressed the problem of an ARC-coated sphere illuminated by an aplanatic beam. The ARC can reduce the scattering force when the sphere is located around the beam focus, so that the high dielectric spheres can be trapped close to the beam focus. This is especially useful when the particle size is large enough so that its curvature approaches that of the planar case. For small particles, the increase in size after coating with the ARC also leads to an enhancement in the optical force. Consequently, in agreement with previous studies, the trap stiffness is significantly enhanced. First, the gradient force increases as a consequence of the increase in particle size after coating. Second, the reduction in the forward scattering force due to the suppression of the backward scattering allows the particle to be trapped closer to the focus, where the transverse gradient force is stronger. The former effect is more significant for smaller spheres, while the latter is more significant for lager spheres. We have also analytically studied the scattering properties of the ARC-coated sphere, and found that its low order Mie coefficients satisfy the generalized Kerker condition while its high order ones do not. So the backward scattering can be suppressed only when an aplanatic beam incidents. This finding not only reveals the mechanism of the ARC using a rigorous wave scattering theory, but also may inspire the use of ARC-coated spheres in manipulating specific light fields. Finally, we remark that the reduced scattering force might also improve optical manipulation in whatever other areas where a conservative optical trap is needed.

## Method

### Derivation of the backward scattering intensity

The normalized scattering intensity can describe the scattering features of a particle illuminated by a beam^37,38^:

8 $$S(\theta ,\varphi )=\mathop{\mathrm{lim}}\limits_{kr\to \infty }{(kr)}^{2}\frac{|{{\bf{E}}}_{s}(r,\theta ,\varphi ){|}^{2}}{|{E}_{0}{|}^{2}},$$

where (*r*, *θ*, *ϕ*) is the spherical coordinate system concentric with the particle, *k* is the wavenumber in the medium, and **E**_*s*_(*r*, *θ*, *ϕ*) is the scattered electric field. The incident and scattered fields can be written in vector spherical wave functions as^37^

9 $$\begin{array}{c}{{\bf{E}}}_{i}({\bf{r}})=\sum _{mn}{i}^{n+1}{E}_{0}[{p}_{mn}{{\bf{N}}}_{mn}^{(1)}(k,{\bf{r}})+{q}_{mn}{{\bf{M}}}_{mn}^{(1)}(k,{\bf{r}})],\\ {{\bf{E}}}_{s}({\bf{r}})=\sum _{mn}{i}^{n+1}{E}_{0}[{a}_{mn}{{\bf{N}}}_{mn}^{(3)}(k,{\bf{r}})+{b}_{mn}{{\bf{M}}}_{mn}^{(3)}(k,{\bf{r}})],\end{array}$$

where *p*_*mn*_ and *q*_*mn*_ are the beam shape coefficients^31–33^, *a*_*mn*_ and *b*_*mn*_ are scattering coefficients given by^37^: *a*_*mn*_ = *a*_*n*_*p*_*mn*_ and *b*_*mn*_ = *b*_*n*_*q*_*mn*_, and ${{\bf{N}}}_{mn}^{(J)}$ and ${{\bf{M}}}_{mn}^{(J)}$ are the vector spherical wave functions given by

$$\begin{array}{rcl}{{\bf{N}}}_{mn}^{(J)}(k,{\bf{r}}) & = & [{\tau }_{mn}(\cos \,\theta ){{\bf{e}}}_{\theta }+i{\pi }_{mn}(\cos \,\theta ){{\bf{e}}}_{\varphi }]\frac{{\xi }_{n}\text{'}(kr)}{kr}{e}^{im\varphi }\\  &  & +\,{{\bf{e}}}_{r}n(n+1){C}_{mn}{P}_{n}^{m}(\cos \,\theta )\frac{{\xi }_{n}(kr)}{{(kr)}^{2}}{e}^{im\varphi },\\ {{\bf{M}}}_{mn}^{(J)}(k,{\bf{r}}) & = & [i{\pi }_{mn}(\cos \,\theta ){{\bf{e}}}_{\theta }-{\tau }_{mn}(\cos \,\theta ){{\bf{e}}}_{\varphi }]\frac{{\xi }_{n}(kr)}{kr}{e}^{im\varphi },\end{array}$$

where ${C}_{mn}=\sqrt{\frac{(2n+1)}{n(n+1)}\frac{(n-m)!}{(n+m)!}}$, *ξ*_*n*_(*kr*) are the Ricatti-Bessel functions, which are *ξ*_*n*_(*kr*) = (*kr*)*j*_*n*_(*kr*) when *J* = 1 and ${\xi }_{n}(kr)=(kr){h}_{n}^{(1)}(kr)$ when *J* = 3, and ${P}_{n}^{m}(\cos \,\theta )$ is the associated Legendre function of the first kind:

10 $${P}_{n}^{m}(x)=\frac{1}{{2}^{n}n!}{(1-{x}^{2})}^{m/2}\frac{{d}^{n+m}}{d{x}^{n+m}}[{({x}^{2}-1)}^{n}],\,\,{P}_{n}^{-m}(x)={(-1)}^{m}\frac{(n-m)!}{(n+m)!}{P}_{n}^{m}(x).$$

The two auxiliary angular functions ${\pi }_{mn}(\cos \,\theta )$ and ${\tau }_{mn}(\cos \,\theta )$ are defined as ${\pi }_{mn}(\cos \,\theta )={C}_{mn}\frac{m}{\sin \,\theta }{P}_{n}^{m}(\cos \,\theta ),$ ${\tau }_{mn}(\cos \,\theta )={C}_{mn}\frac{d}{d\theta }{P}_{n}^{m}(\cos \,\theta ),$

which satisfy^44^

11 $${\pi }_{-mn}(x)={(-1)}^{m+1}{\pi }_{mn}(x),\,{\tau }_{-mn}(x)={(-1)}^{m}{\tau }_{mn}(x).$$

For a linearly polarized Gaussian beam, the beam shape coefficients can be written down analytically for a particle located on the beam axis^39^:

12 $$\begin{array}{c}{p}_{1n}={g}_{n}=\tfrac{\sqrt{2n+1}}{2{i}^{n}}[\tfrac{(n+1)i}{2n+1}{j}_{n-1}(ik{z}_{c})+{j}_{n}(ik{z}_{c})-\tfrac{ni}{2n+1}{j}_{n+1}(ik{z}_{c})]\tfrac{k{l}_{0}}{{e}^{k{l}_{0}}},\\ \,\,\,\,\,\,{p}_{1n}=-{p}_{-1n}={q}_{1n}={q}_{-1n},\\ \,\,\,\,\,\,{p}_{mn}={q}_{mn}=0,\,m\ne \pm 1.\end{array}$$

where ${l}_{0}=1/2k{w}_{0}^{2}$ is the Rayleigh diffraction length with *w*_0_ being the waist radius, and *z* = *l*_0_ − *iz*_0_ with *z*_0_ being the location of the beam center. As one can see from Eq. (12), for both *p*_*mn*_ and *q*_*mn*_, only the azimuthal modes with *m* = ±1 contribute. Then, the scattered field in the far field (*kr* → ∞) is given by

13 $$\begin{array}{rcl}\mathop{\mathrm{lim}}\limits_{kr\to \infty }({kr}){{\bf{E}}}_{s} & = & \mathop{\mathrm{lim}}\limits_{kr\to \infty }({kr})\sum _{n}{i}^{n+1}{E}_{0}[{a}_{n}({p}_{-1n}{{\bf{N}}}_{-1n}^{(3)}+{p}_{1n}{{\bf{N}}}_{1n}^{(3)})\\  &  & +\,{b}_{n}({q}_{-1n}{{\bf{M}}}_{-1n}^{(3)}+{q}_{1n}{{\bf{M}}}_{1n}^{(3)})]\\  & = & \mathop{\mathrm{lim}}\limits_{kr\to \infty }({kr})\sum _{n}{i}^{n+1}{E}_{0}\,{g}_{n}[{a}_{n}(\,-\,{{\bf{N}}}_{-1n}^{(3)}+{{\bf{N}}}_{1n}^{(3)})\,\\  &  & +\,{b}_{n}({{\bf{M}}}_{-1n}^{(3)}+{{\bf{M}}}_{1n}^{(3)})]\\  & = & \mathop{\mathrm{lim}}\limits_{kr\to \infty }2\sum _{n}{i}^{n+1}{E}_{0}{g}_{n}\\  &  & \times \,[\begin{array}{c}{a}_{n}([{\tau }_{1n}(x)\cos \,\varphi {{\bf{e}}}_{\theta }-{\pi }_{1n}(x)\sin \,\varphi {{\bf{e}}}_{\varphi }]{\xi ^{\prime} }_{n}(kr)\\ +i{b}_{n}({\pi }_{1n}(x)\cos \,\varphi {{\bf{e}}}_{\theta }-{\tau }_{1n}(x)\sin \,\varphi {{\bf{e}}}_{\varphi }){\xi }_{n}(kr)\end{array}]\end{array}$$

Using the asymptotical formula for the Ricatti-Bessel functions for *kr* → ∞, *ξ*_*n*_(*kr*) ∼ (−*i*)^*n*+1^*e*^*ikr*^, ${\xi ^{\prime} }_{n}$(*kr*) ∼ (−*i*)^*n*^*e*^*ikr*^ = *iξ*_*n*_(*kr*), Eq. (13) reduces to

14 $$\mathop{\mathrm{lim}}\limits_{kr\to \infty }({kr}){{\bf{E}}}_{s}=\mathop{\mathrm{lim}}\limits_{kr\to \infty }2\sum _{n}i{E}_{0}{g}_{n}[\begin{array}{c}{a}_{n}([{\tau }_{1n}(x)\cos \,\varphi {{\bf{e}}}_{\theta }-{\pi }_{1n}(x)\sin \,\varphi {{\bf{e}}}_{\varphi }]\\ +{b}_{n}({\pi }_{1n}(x)\cos \,\varphi {{\bf{e}}}_{\theta }-{\tau }_{1n}(x)\sin \,\varphi {{\bf{e}}}_{\varphi })\end{array}]{e}^{ikr}.$$

For the backward direction, namely *x* = cos*π* = −1, the auxiliary functions read

15 $${\pi }_{1n}=\,-\,{\tau }_{1n}={(-1)}^{n+1}\frac{\sqrt{n+1}}{2}.$$

Then, substituting Eqs (14) and (15) into Eq. (8), we have the backward scattering intensity as

16 $$S(\theta ,\varphi )={|\sum _{n}(n+1){g}_{n}({a}_{n}-{b}_{n})(\cos \varphi {{\bf{e}}}_{\theta }-\sin \varphi {{\bf{e}}}_{\varphi })|}^{2},$$

which is Eq. (3).
